# Elevated Serum Chloride Levels Contribute to a Poor Prognosis in Patients with IgA Nephropathy

**DOI:** 10.1155/2021/3598135

**Published:** 2021-10-08

**Authors:** Yaling Zhai, Xingchen Yao, Yuanyuan Qi, Jingge Gao, Yazhuo Chen, Xinnian Wang, Feng Wu, Zhanzheng Zhao

**Affiliations:** ^1^Department of Nephrology, The First Affiliated Hospital of Zhengzhou University, Zhengzhou, China; ^2^The Renal Research Institution of Zhengzhou University, Zhengzhou, China

## Abstract

**Introduction:**

The identification of reliable prognostic factors is a crucial requirement for patients with IgA nephropathy (IgAN). Here, we explored the relationship between serum chloride levels and prognosis in patients with IgAN.

**Methods:**

We recruited all patients with primary IgAN, as diagnosed by renal biopsy, between 1^st^ January 2015 and 1^st^ April 2019. Patients were divided two groups (high chloride group and low chloride group) based on the best cut-off values from survival receiver operating characteristic (ROC) curves. The baseline clinicopathological characteristics of two groups were then compared. Cox proportional hazard models were used to determine the prognostic value of serum chloride levels in patients with IgAN. Finally, we screened reliable prognostic indicators and built a clinical prediction model and validated the performance of the model.

**Results:**

Compared with patients in the high chloride group, patients in the low chloride group had significantly lower levels of 24-hour urinary total protein (24 h-UTP), serum creatinine (sCr), and higher levels of hemoglobin (Hb), albumin (all *p* < 0.05), and less proportion of Oxford classification grade E1 (endothelial cell proliferation) and T2 (renal tubule atrophy or renal interstitial fibrosis). Cox analysis revealed that serum chloride level ≥ 105.4 mmol/L was a significant and independent risk factor for prognosis in patients with IgAN (*p* < 0.05). Serum chloride, sCr, T, hypertension, and Hb were used to generate a predictive model for prognosis. The*c*-indices of our predictive model were 0.80, 0.86, and 0.78, for 1, 2, and 3 years, respectively; Brier scores were 0.06, 0.09, and 0.16, respectively.

**Conclusions:**

A serum chloride level ≥ 105.4 mmol/l was identified as a significant and independent risk factor for the prognosis of patients with IgAN. A predictive prognosis model was generated using serum chloride, sCr, T, hypertension, and Hb; this model exhibited a good predictive effect.

## 1. Introduction

Primary IgA nephropathy (IgAN) is the most common type of idiopathic glomerulonephritis in the world and the main cause of end-stage renal disease (ESRD) in patients with primary glomerular disease [1]. At present, the most accepted pathogenic mechanism underlying IgAN relates to the “multiple hits” theory [2], “Hit-1,” production of galactose-deficient IgA1 (Gd-IgA1) in circulation, “Hit-2,” production of antibodies against Gd-IgA1, “Hit-3,” formation of Gd-IgA1 containing circulating immune complex (CIC), and “Hit-4,” deposition of CIC in kidney that contributes to renal injury. The important role of immunological and inflammatory mechanisms in the occurrence and development of IgAN has gradually become widely recognized [2–4]. Moreover, researchers have gradually identified a number of predictors for the prognosis of patients with IgAN based on clinical, pathological, genetic, and noninvasive biological markers [5]. However, these parameters are associated with poor sensitivity, trauma, and high costs. Therefore, there is a clear need to identify a more convenient and accurate predictor of prognosis for patients with IgAN.

The progressive loss of the kidney function is inevitably associated with a number of electrolyte and acid-based alterations [6–9]. Most of these disorders are intricately linked to the morbidity and mortality of patients with kidney disease [10], particularly with regards to potassium imbalance, metabolic acidosis, and the dysregulation of bone mineral metabolism. The chloride ion is the most abundant anion in the extracellular fluid and plays a crucial role in regulating a number of functions in the human body [11], including the maintenance of osmotic pressure, acid-based balance, muscular activity, and the movement of water between fluid compartments [11]. Over time, studies have increasingly begun to recognize the importance of chloride, especially with ongoing investigations relating to acid-base mechanisms [12] and chloride ion channels [13]. However, very few studies have investigated the relationship between serum chloride and the renal prognosis of IgAN. Based on previous studies, serum chloride disorders may exert an adverse effect on renal function; it is currently believed that hyperchloremia is more closely related to the severity of kidney injury than hypochloremia [14]. Higher levels of serum chloride can cause the release of thromboxane [15] and enhance the response of renal vasoconstrictors, such as angiotensin II [16]. In addition, high chloride levels can induce a glomerular feedback mechanism in dense plaques, thus causing the contraction of afferent arterioles and the glomerular mesentery; this process may also reduce glomerular filtration rate [17]. In addition, high chloride levels are often associated with metabolic acidosis [12]. Perchloric acidosis increases the production of endothelin-1 and aldosterone, thus leading to tubulointerstitial inflammation and injury, thereby accelerating the progression of chronic kidney disease (CKD) [10]. Therefore, we hypothesized that serum chloride levels will affect the clinical and pathological indicators and prognosis of IgAN. The main objective of this study was to explore the relationship between serum chloride and the clinical and pathological indicators of IgAN, as well as the prognosis of patients with this condition.

## 2. Materials and Methods

### 2.1. Study Population

We enrolled patients with a biopsy-based diagnosis of primary IgAN between 1^st^ January 2015 and 1^st^ April 2019 in the First Affiliated Hospital of Zhengzhou University. The inclusion criteria included the following items: (1) primary IgAN was diagnosed by renal biopsy, (2) the follow-up time was more than 6 months, and (3) no glucocorticoid or immunosuppressants were used before renal biopsy and (4) an initial estimated glomerular filtration rate (eGFR) ≥ 15 mL/min/1.73 m^2^ at the time of renal biopsy. The following exclusion criteria were applied: (1) patients with secondary IgAN, including those with chronic hepatitis B, Henoch-Schonlein purpura, ankylosing spondylitis, systemic lupus erythematosus, and rheumatoid arthritis; (2) patients for whom the number of glomeruli in renal biopsy specimens was <10; (3) patients who did not have a complete set of clinicopathological data; (4) patients with an eGFR < 15 mL · min^−1^ (1.73 m^2^)^−1^; (5) patients with acute kidney failure at the time of renal biopsy; and (6) patients who had used choride-containing fluids before renal biopsy.

### 2.2. Acquisition of Clinical, Biochemical, and Histopathological Data

Baseline clinicopathological data were recorded at the time of the renal biopsy. The Oxford classification was used to score each patient's condition; this involved two renal pathologists who were blinded to the clinical data. First, the data were independently diagnosed by a junior nephrology pathologist and then reviewed by a senior nephrology pathologist. If these physicians had different opinions, the diagnosis of a senior nephrologist was prevail [18]. The mesangial cell proliferation score (M) was defined as M0 if the score was ≤0.5 and M1 if the score was>0.5. Endothelial cell hyperplasia (E) and segmental sclerosis or adhesion (S) were defined as E0 and S0 if absent and E1 and S1 if present. Renal tubule atrophy or renal interstitial fibrosis (T) was defined as T0 if 0% -25% of the cortical area was associated with renal tubule atrophy or renal interstitial fibrosis, T1 for 26% -50%, and T2 for >50%. Crescentic lesions (C) were defined as C0 if there were no crescents, C1 for <25% globular crescents, or C2 for ≥25% globular crescents. The degree of vascular injury (A) was defined as A0 if there were no obvious abnormalities, A1 if there was vascular wall thickening, and A2 if there was vascular wall thickening and other lesions, such as fibrinoid necrosis and vitreous degeneration. Renal tubular necrosis was scored as 0 if absent and 1 if present. Immunofluorescence results relating to immunoglobulin G (IgG), immunoglobulin M (IgM), immunoglobulin A (IgA), and glomerular C3 were scored as follows: 0 represents -/+, 1 represents +, 2 represents ++, 3 represents +++, and 4 represents ++++.

Serum creatinine (sCr), serum urea nitrogen (BUN), uric acid (UA), 24-hour urinary total protein (24 h-UTP), serum complements 3(sC3) and 4 (sC4), and serum immunoglobulins were detected by a sarcosine oxidase enzymatic method, a urease method, a urase-peroxidase coupling method, a biuret method, and an immunoturbidimetry method, respectively, using a Roche Cobas 8000c702 Automatic Biochemical Analyzer. Albumin and electrolytes were detected by bromocresol green colorimetry and an indirect ion selective electrode method, respectively, using a Roche Cobas 8000c701 Automatic Biochemical Analyzer. Hemoglobin was detected by a sodium dodecyl sulfate method using a Beckman Coulter LH750 Blood Cell Analyzer.

In order to analyze prognosis, we used an established definition for the composite endpoint: a doubling in sCr level when compared to baseline data, ESRD, death, or a decline in eGFR that was >30% [19]. ESRD was defined as when the eGFR was ≤15 mL/min per 1.73 m^2^ or when the patient required renal replacement therapy(RRT) (including hemodialysis, peritoneal dialysis, or renal transplantation).

### 2.3. Statistical Methods

The SurvivalROC package in *R* software (version 3.6.3; http://www.R-project.org) was used to calculate the best cut-off point for serum chloride. Normally distributed quantitative variables were expressed as means ± standard deviations (SDs), and based on the homogeneity of variance, the *t*-test or corrected *t*-test was used for comparisons between groups. Nonnormally distributed features were expressed as medians and interquartile ranges, and the Wilcoxon rank sum test was used for comparisons between groups. Categorical variables were expressed by frequency and percentage, and the chi-square test was used for comparison between groups. Kaplan-Meier survival curves, along with unadjusted and adjusted Cox proportional hazards models, were used to analyze the relationship between serum chloride level and the prognosis of patients with IgAN. Age, sex, hypertension, 24 h-UTP, sCr, and Oxford classification scores were adjusted in multivariable-adjusted Cox proportional hazards models [20–23]. Furthermore, we built a predictive model for prognosis. Initial clinicopathological variables associated with survival were evaluated using univariate Cox regression (Supplemental Material Table 1). Then, we used forward-backward selection with the Akaike information criterion (AIC) to identify the final predictive factors. On the basis of this analysis, we used serum chloride, hypertension, sCr, hemoglobin (Hb), and Oxford classification grade T, in our final predictive model. A nomogram was then created to present the model in a visual manner. The internal stability of the model was then tested using the bootstrap approach. The model performance was evaluated based on the predictive accuracy for individual outcomes (discriminating ability) and the accuracy of point estimates of the survival function (calibration). SPSS version 21 software (SPSS Inc., Chicago, IL, USA) and *R* software (version 3.6.3, http://www.R-project.org) were used for statistical analysis. *p* < 0.05 was considered to be statistically significant.

## 3. Results

### 3.1. Demographic and Clinicopathological Characteristics

Between 1^st^ January 2015 and 1^st^ April 2019, we identified 394 patients with primary IgAN whose follow-up time exceeded 6 months. The clinical and pathological characteristics at baseline are summarized in Table 1. In total, 212 (54%) patients were male, and the mean age at biopsy was 35 ± 12 years, 154 (39%) patients had hypertension, the mean serum chloride level was 104.10 ± 3.47 mmol/l, the sCr was 85.25 (68.00, 120.00) umol/L, and 24 h-UTP was 1.86 (0.95,4.04) g/d. In Oxford classification, 91 (23%) patients were in the M1 group, 117 (30%) patients were in the E1 group, and 253 (64%) patients were in the S1 group, patients count with T0, T1, and T2 that was 275 (70%), 52 (13%), 67 (17%), and C0, C1, and C2 were 246 (63%),135 (34%), and 12 (3%), respectively. Patients were followed for 14 (9, 24 months) months, and 46 patients reached the composite endpoint.

### 3.2. Experimental Grouping Based on Serum Chloride Levels

**Figure 1:**
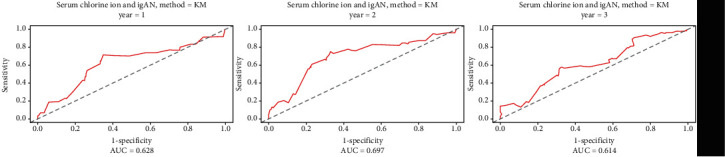
Survival receiver operating characteristic (ROC) curves for 1-, 3-, and 3-year survival. The areas under the receiver operating characteristics curves (AUCs) were 0.63, 0.70, and 0.61 for 1-, 2-, and 3-year follow-up, respectively. The cut-off values were 105.50 for 1-year follow-up and 105.40 for 2- and 3- ear follow-up.

According to survivalROC, the areas under the receiver operating characteristic curves (AUCs) for 1 year, 2 years, and 3 years were 0.63, 0.70, and 0.61, respectively; the best cut-off points were 105.50 for 1 year and 105.40 mmol/l for 2 years and 3 years (shown in Figure 1). The patients were divided into two groups (a low chloride group and a high chloride group) according to the cut-off value of 105.40 mmol/L. There were no significant differences in subsequent analyses regardless of whether the grouping was based on a cut-off of 105.40 mmol/L or 105.50 mmol/L. There were 247 patients in the low chloride group and 159 patients in the high chloride group.

### 3.3. Patients with Higher Serum Chloride Levels Had More a Severe Clinical and Pathological Presentation

The clinical and pathological characteristics of the 394 patients in the low chloride group and the high chloride group are shown in Table 1. The 24 h-UTP and sCr in the low chloride group were significantly lower than in the high chloride group [24-UTP: 1.46 (0.84, 3.44) vs. 2.64 (1.28, 4.98) g, *p* < 0.001; sCr: 83.00 (67.00, 111.25) vs. 94.50 (70.00, 145.00) *μ*mol/l, *p* = 0.004]. Serum albumin and Hb levels in the low chloride group were significantly higher than those in the high chloride group [serum albumin: 39.10 (34.30, 42.90) vs. 33.80 (24.95, 38.95) g/l, *p* < 0.001; Hb: 130.40 ± 19.30 vs. 125.20 ± 19.10, *p* = 0.010]. In addition, we found that serum urea nitrogen, sC3, serum sodium, serum calcium, serum magnesium, and serum immunoglobulin G also showed significant differences when compared between the two groups. No difference between the two groups existed in other indicators including hypertension, UA, urine red blood cell, sC4, phosphorus, potassium, serum immunoglobulin A, and serum immunoglobulin M.

Furthermore, we also identified significantly fewer patients with an Oxford classification grade of E1 and an Oxford classification grade of T2 in the low chloride group [E: 185 (77)/55 (23) vs. 92 (60)/62 (40), *p* < 0.001; T: 178 (74)/30 (13)/32 (13) vs. 97 (63)/22 (14)/35 (23), *p* = 0.012]. there was no significant difference in the rest indicators including Oxford classification grade of M, S, C, and IgG, IgM, IgA, glomerular C3, A, tubular necrosis, and glomerular sclerosis, as well as segmental sclerosis.

### 3.4. Serum Chloride Level Was Able to Predict the Prognosis of Patients with IgAN

**Figure 2:**
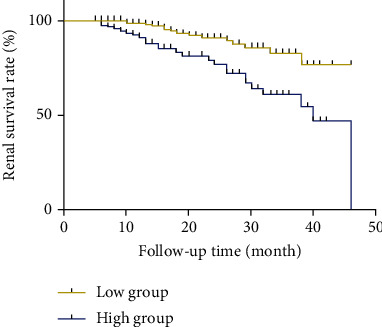
Kaplan-Meier renal survival curves for patients with IgA nephropathy (IgAN) according to serum chloride levels. Patients with IgAN were classified into two groups according to serum chloride levels: a low chloride group (<105.4 mmol/L, red line) and a high chloride group (≥105.4 mmol/L, blue line). Log-rank rest revealed that the cumulative renal survival rates in the high chloride group were significantly worse than those in the low chloride group (*p* < 0.001).

In order to investigate the relationship between serum chloride level and the prognosis of patients with IgAN, we first plotted a Kaplan-Meier renal survival curve for patients with IgAN according to serum chloride levels; results indicated that patients in the low chloride group had a significantly better renal survival rate than those in the high chloride group (*p* < 0.001) (Figure 2). Then, we performed unadjusted Cox regression analyses with no other adjusted factor except for serum chloride. These analyses showed that a significantly higher number of patients in the high chloride group reached the composite end point (hazard ratio (HR), 3.22; 95% confidence interval (95% CI), 1.76-5.86, *p* < 0.001) (Table 2). Then, we applied three adjusted Cox proportional hazards models. Our reasons for choosing these indicators were as follows. Firstly, in clinical research, age and gender need to be adjusted initially in the Cox proportional hazards model, irrespective of whether there are significant differences or not [20–23]. Secondly, hypertension, 24 h-UTP, sCr, and Oxford classification scores have been proven to be vital risk factors for the prognosis of patients with IgAN [20–23]. When adjusted for sex, age, sCr, 24 h-UTP, hypertension, and Oxford classification scores, a serum chloride level ≥ 105.4 mmol/L still showed a significant association with a poor renal outcome in patients with IgAN (model 1: HR, 3.09; 95% CI, 1.69-5.64, *p* < 0.001; model 2: HR, 2.33; 95% CI, 1.20-4.55, *p* = 0.01; model 3: HR, 2.05; 95% CI, 1.03-4.07, *p* = 0.04). These results revealed that a serum chloride level ≥ 105.40 mmol/l was an independent risk factor for the prognosis of patients with IgAN (Table 2).

### 3.5. Generation of a Predictive Prognostic Model Based on Clinical and Pathological Parameters and Internal Validation

**Figure 3:**
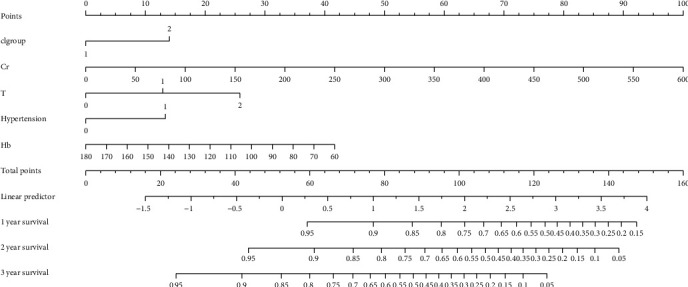
A nomogram predicting the probabilities of 1-, 2-, and 3-year renal survival. We built a nomogram based on the prognostic model (including sCr, hypertension, T, Hb, and serum chloride (<105.40 mmol/L or ≥105.40 mmol/L)). Higher total scores based on the sum of the assigned number of points for each factor in the nomogram were associated with a worse prognosis.

**Figure 4:**
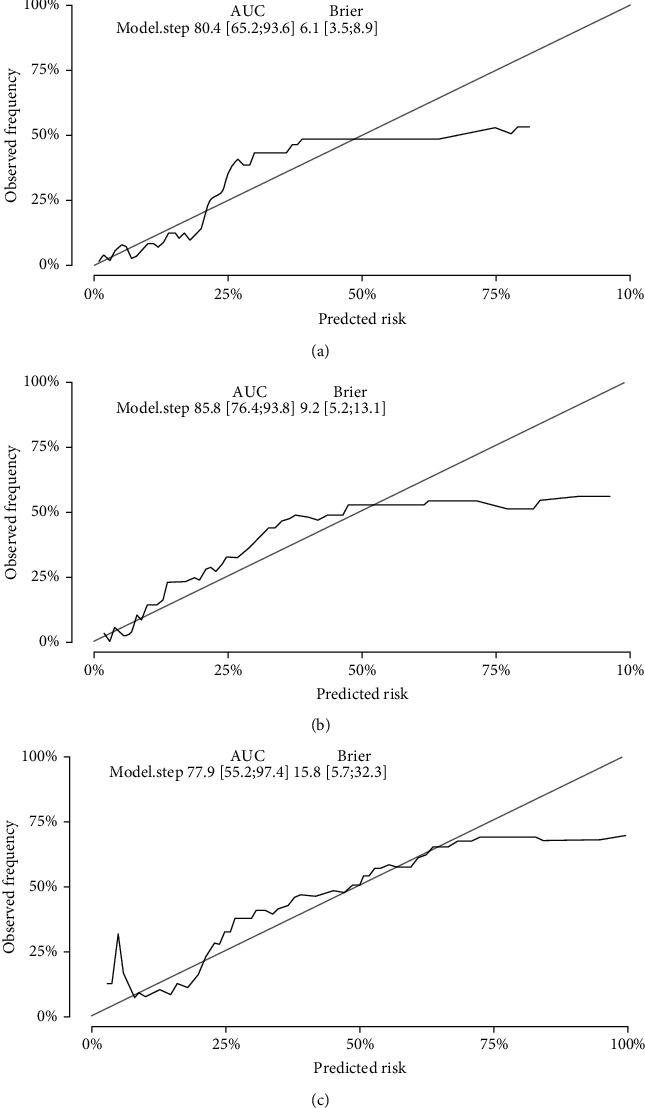
*c*-indices and calibration curves for the new model. 200 sample bootstrapped calibration plots for the prediction of 1–3-year renal survival are shown. The gray line represents the ideal fit while the black line represents practical fit. The *c*-indices for 1-3 years were 0.80 (95% CI: 0.65-0.94), 0.86 (95% CI: 0.77-0.94). and 0.78 (95% CI: 0.55-0.97), respectively. Brier scores were 0.06 (95% CI: 0.04-0.09), 0.09 (95% CI: 0.05-0.13). and 0.16 (95% CI: 0.06-0.32), respectively.

According to univariate Cox regression analysis, we incorporated serum chloride level (<105.40 mmol/L or ≥105.40 mmol/L), hypertension (yes or no), sCr, UA, 24 h-UTP, serum phosphorus, Hb, and Oxford classification grade (T, M, S, and A) as our initial prognostic features (Supplemental Material Table 1). Then, these parameters were reduced to the five most useful potential predictors: serum chloride (<105.40 mmol/L or ≥105.40 mmol/L), sCr, Oxford classification grade T, hypertension, and Hb using forward-backward selection with the AIC. A nomogram based on the prognosis model was then constructed to estimate 1-, 2- and 3-year renal survival (shown in Figure 3). Then, bootstrap validation, with 200 re-tests, was employed for internal validation. The discriminative ability of the final model was then assessed using *C* statistics. The *c*-index of this model was 0.80 (95% CI 0.65-0.94) for 1 year, 0.86 (95% CI 0.77-0.94) for 2 years, and 0.78 (95% CI 0.55-0.97) for 3 years. The calibration was evaluated using calibration plots and Brier scores. The Brier score for this model was 0.06 (95% CI 0.04-0.09) for 1 year, 0.09 (95% CI 0.05-0.13) for 2 years, and 0.16 (95% CI 0.06-0.32) for 3 years; calibration plots are shown in Figure 4.

## 4. Discussion

In the present study, we demonstrated that a serum chloride level ≥ 105.4 mmol/L at the time of renal biopsy was associated with more severe clinical and pathological manifestation and worse renal outcomes.

The early identification or prediction of a poor prognosis in patients with IgAN is often very difficult but remains a critical requirement. In recent years, an increasing number of prognostic indicators for IgAN have been investigated [24]. Of these, hypertension, massive proteinuria, renal impairment, albumin, and severe histological findings have been widely accepted [25]. However, these indicators are also associated with some disadvantages, such as limited sensitivity, trauma, or high costs. Over recent years, there has been significant interest in the investigation of biomarkers and genetic indicators [2]; however, these indicators can be relatively expensive and are therefore likely to create an economic burden for some patients. Therefore, there is a clear need to identify a convenient, cheap, specific, and highly sensitive indicator.

In a previous study, Mary et al. demonstrated that hypertonic NaCl in dogs led to transient renal vasodilation that was probably related to plasma hypertonicity followed by sustained renal vasoconstriction and reduced eGFR [15]. In another study, Tanake et al. used stroke-prone spontaneously hypertensive rats (SHRSP) to demonstrate that chloride ions amplified microangiopathy by exacerbating hypertension and potentially by increasing plasma renin activity (PRA), potentially by constricting the renal afferent arteriole [16]. In addition, chloride ions can induce a glomerular feedback mechanism in dense plaques that involves the contraction of afferent arterioles and the glomerular mesentery; this process can also reduce the glomerular filtration rate [26]. In addition, a high level of chloride may not only influenced IgAN by itself, and it may also be accompanied by metabolic acidosis, a process that is also known to cause damage to renal function [11]. Evidence to support a relationship between metabolic acidosis and the prognosis of patients with IgAN remains limited; although, metabolic acidosis has been associated with accelerated CKD progression and elevated all-cause mortality, thus providing new clues for the pathogenesis of IgAN [27].

In the present study, we used survivalROC software to divide our patients into two groups according to chloride levels. Although variable statistical methods are available for cut-off point selection [24], these tend not to relate to cut-off points for disease prognosis and early prediction, thus reducing clinical applicability and reducing statistical power [28]. Previous research has revealed that survivalROC software can facilitate the development of eligibility criteria for clinical trials [28]; this meant that our cut-off value was not only useful for experimental grouping but they were also useful for the early prediction of disease and prognosis.

When comparing the demographic, clinical, and pathological data between the two groups at baseline, we found that the clinical and pathological indicators of IgAN were more serious when the serum chloride level was ≥105.4 mmol/L.

According to univariate and multivariate Cox regression analyses, a serum chloride level ≥ 105.4 mmol/L was an independent and significant risk factor for IgAN. Although the range for serum chloride levels in the clinical laboratory dictates a higher limit of 106-107 mmol/L, we specifically considered the relationship between serum chloride level and the prognosis of patients with IgAN. Therefore, we believe that the higher limit of serum chloride level in such patients should be 105.4 mmol/L. This finding suggested that clinicians should pay close attention to changes in serum chloride level when treating patients with IgAN and to ensure that the level remains below 105.4 mmol/L.

In addition, we also established a clinical model for predicting prognosis that included sCr, serum chloride level (<105.4 mmol/l or ≥105.4 mmol/l), hypertension, Oxford classification grade T, and Hb. Our predictive model for prognosis exhibited a good discriminative ability according to *c*-index. However, the calibration plots did not demonstrate very good agreement.

Next, we considered our findings in relation to a number of factors. Firstly, 0.9% NaCl solution is widely used in clinical work; this contains a concentration of chloride ions that is higher than the normal physiological range [29]. This could cause hyperchloremia and metabolic acidosis [30]. Considering the influence of serum chloride level on IgAN, it might be possible to replace NaCl solution with other solutions or restrict the use of NaCl solution; these possibilities should be investigated further. However, some articles in the existing literature may support our suggestions [17, 30]. For example, Malley et al. used a double-blinded method to compare the outcomes of renal transplantation patients involving lactated Ringer's solution and 0.9% NaCl solution [30]. Data showed that hyperchloremia and metabolic acidosis were more common in the group of patients receiving 0.9%NaCl. In another study, Yunos et al. proved that restricting the use of chlorine-rich fluids in a tertiary ICU reduced the incidence of acute kidney injury and renal replacement therapy requirements [30]. It is important to highlight that the blood samples acquired from patients in our study were collected on the morning of the day of renal biopsy before the use of any chlorine-containing fluids, thus indicating that our results were not influenced by confounding factors. Secondly, 99.1% of chloride ions are reabsorbed by the kidney [26]. It may be possible to improve the prognosis of IgAN patients with high serum chloride levels by reducing the reabsorption of chloride ions in the kidney. Further research is now required to identify pathways that could inhibit the reabsorption of chloride ions. There are many mechanisms related to the reabsorption of chloride ions in the kidney [11, 31]. Of these, the most important process occurs in the latter half of the proximal tubule, where chloride ions are transported into cells via the Cl^−^ -HCO_3_^−^ exchanger located on the top membrane of the epithelial cell; chloride ions that enter the cell are then transported to the intercellular fluid via the K^+^-Cl^−^ symporter on the basilar membrane and then absorbed into the blood. In addition, chloride ions can also be reabsorbed via a paracellular pathway [31]. Therefore, it may be possible to inhibit the reabsorption of chloride ions by inhibiting the transmembrane transport of chloride ions in the proximal renal tubules or the paracellular pathway, thereby reducing the serum levels of chloride and improving the prognosis of patients with IgAN. However, this possibility needs to be investigated further.

Our study has certain limitations that need to considered. First, this was a retrospective study carried out in a single-center. Second, we did not investigate the mechanisms underlying the elevation of serum chloride levels. In addition, our study has not been validated in other races.

## 5. Conclusions

In conclusion, serum chloride level ≥ 105.4 mmol/L is an independent and significant risk factor for the prognosis of patients with IgAN. These findings indicate that we should pay more attention to serum chloride levels in patients with IgAN.

## Figures and Tables

**Table 1:** Relationship between the serum chloride and demographic, clinical, and pathologic data.

Characters	Total (*n* = 394)	Low group (*n* = 247)	High group (*n* = 159)	*p* value
Demographic data				
Age (years)	35 ± 12	34 ± 12	36.±12	0.050
Male (*n*%)	212 (54)	125 (52)	87 (57)	0.392
Clinical data				
Hypertension (*n*%)	154 (39%)	89 (36%)	65 (41%)	0.309
BUN (mmol/l)	5.80 (4.50, 7.70)	5.62 (4.40, 7.15)	6.20 (4.62, 8.31)	0.043
sCr (umol/l)	85.25 (68.00, 120.00)	83.00 (67.00, 111.25)	94.50 (70.00, 145.00)	0.004
UA (umol/l)	341.00 (283.00,422.00)	329.00 (271.00, 415.00)	369.00 (293.75, 427.25)	0.093
ALB (g/l)	37.30 (29.20, 41.50)	39.10 (34.30, 42.90)	33.80 (24.95, 38.95)	<0.001
24 h-UTP (g/d)	1.86 (0.95,4.04)	1.46 (0.84,3.44)	2.64 (1.28,4.98)	<0.001
Urine RBC (/HP)	42.00 (9.00,181.00)	41.00 (10.00,161.00)	49.00 (8.00,187.00)	0.730
Serum C3 (g/l)	1.16 (0.93,1.41)	1.20 (0.98,1.48)	1.11 (0.90,1.26)	0.004
Serum C4 (g/l)	0.26 (0.21,0.32)	0.25 (0.21,0.31)	0.28 (0.21,0.33)	0.124
Hb (g/l)	128.40 ± 19.40	130.40 ± 19.30	125.20 ± 19.10	0.010
K (mmol/l)	4.42 ± 0.49	4.38 ± 0.45	4.47 ± 0.55	0.091
Na (mmol/l)	143.00 ± 3.00	142.00 ± 3.00	144.00 ± 3.00	<0.001
Ca (mmol/l)	2.20 ± 0.17	2.24 ± 0.16	2.14 ± 0.18	<0.001
Mg(mmol/l)	0.94 ± 0.11	0.95 ± 0.10	0.93 ± 0.11	0.016
P (mmol/l)	1.24 ± 0.24	1.24 ± 0.22	1.24 ± 0.26	0.812
SIgA (g/l)	2.89 (2.12,3.88)	2.86 (2.17,3.95)	2.89 (1.97,3.75)	0.459
SIgG (g/l)	9.69 (6.57,11.80)	10.20 (7.99,12.80)	8.63 (5.32,10.97)	0.009
SIgM (g/l)	1.01 (0.76,1.43)	1.06 (0.83,1.40)	0.91 (0.66,1.50)	0.137
Histopathological data				
M [M0/M1, *n* (%)]	303 (77)/91 (23)	192 (80)/48 (20)	111 (72)/43 (28)	0.069
E [E0/E1, *n* (%)]	277 (70)/117 (30)	185 (77)/55 (23)	92 (60)/62 (40)	<0.001
S [S0/S1, *n* (%)]	141 (36)/253 (64)	87 (36)/153 (64)	54 (35)/100 (65)	0.811
T [T0/T1/T2, *n* (%)]	275 (70)/52 (13)/67 (17)	178 (74)/30 (13)/32 (13)	97 (63)/22 (14)/35 (23)	0.012
C [C0/C1/C2, *n* (%)]	246 (63)/135 (34)/12 (3)	155 (65)/78 (33)/6 (3)	91 (59)/57 (37)/6 (4)	0.227
IgG [0/1/2, *n* (%)]	325 (83)/50 (13)/18 (5)	195 (82)/33 (14)/11 (5)	130 (84)/17 (11)/7 (5)	0.491
IgM (0/1/2/3, *n* (%)]	115 (29)/200 (51)/66 (17)/4 (1)	70 (30)/123 (53)/38 (16)/3 (1)	45 (30)/77 (51)/28 (18)/1 (0.7)	0.835
IgA [0/1/2/3/4, *n* (%)]	4 (1)/22 (6)/277 (71)/88 (22)/1 (0.3)	2 (0.8)/10 (4)/168 (71)/57 (24)/1 (0.4)	2 (1)/12 (8)/109 (71)/31 (20)/0 (0)	0.133
C3 [0/1/2/3, *n* (%)]	108 (28)/82 (21)/165 (42)/33 (9)	62 (26)/55 (23)/100 (42)/19 (8)	46 (31)/27 (18)/65 (42)/14 (9)	0.909
A [0/1/2, *n* (%)]	133 (34)/83 (21)/178 (45)	90 (38)/46 (19)/104 (43)	43 (28)/37 (24)/74 (48)	0.131
Tubular necrosis [0/1, *n* (%)]	37 (95)/19 (5)	229 (95)/11 (5)	146 (95)/8 (5)	0.782
GS%	0.10 (0.00,0.27)	0.10 (0.00,0.26)	0.09 (0.00,0.34)	0.707
SS%	0.04 (0.00,0.10)	0.03 (0.00,0.10)	0.04 (0.00,0.11)	0.703

Low group: serum chloride < 105.4 mmol/L and high group: serum choride ≥ 105.4 mmol/L. BUN: blood urine nitrogen; sCr: serum creatinine; UA: urine acid; ALB: albumin; 24 h-UTP: 24hour-urine protein; urine RBC: urine red blood cell; serum C3: serum complement 3; serum C4: serum complement 4; Hb: hemoglobin; K: potassium; Na: sodium; Ca: calcium; Mg: magnesium; P: phosphorus; SIgA: serum immunoglobulin A; SIgG: serum immunoglobulin G; SIgM: serum immunoglobulin M. For Oxford classification, mesangial cell proliferation score (M): M0 for score ≤ 0.5, M1 for score > 0.5; endothelial cell hyperplasia (E): E0 for absent and E1 for present; segmental sclerosis or adhesion (S): S0 for absent and S1 for present; renal tubule atrophy or renal interstitial fibrosis (T): T0 for 25% renal tubule atrophy or renal interstitial fibrosis, T1 for 26% ~ 50% ,and T2 ≥ 50%; crescentic lesions (C): C0 is no crescent, C1 is <25% globular crescent and C2 is ≥25% globular crescent. For immunofluorescence, immunoglobulin G(IgG): 0 for -/+, 1 for +, and 2 for ++; IgM, immunoglobulin M(IgM): 0 for -/+, 1 for +, 2 for ++, and 3 for +++; immunoglobulin A(IgA): 0 for-/+, 1 for +, 2 for ++, 3 for +++, and 4 for ++++; complement 3(C3): 0 for-/+, 1 for +, 2 for ++, and 3 for+++; the degree of vascular injury (A): 0 for no obvious abnormality, 1 for simple vascular wall thickening and 2 for not only vascular wall thickening, but also other lesions, such as fibrinoid necrosis and vitreous degeneration; renal tubular necrosis: 0 for no necrosis and 1 for necrosis. GS: glomerular sclerosis; SS: segmental sclerosis.

**Table 2:** Cox regression analysis of the effect of serum chloride ion on the prognosis of IgAN patients.

Models	HR (95% CI)	*p* value
Unadjusted	3.22 (1.76-5.86)	<0.001
Model1^a^	3.09 (1.69-5.64)	<0.001
Model2^b^	2.33 (1.20-4.55)	0.01
Model3^c^	2.05 (1.03-4.07)	0.04

HR: hazard ratio; 95% CI: 95% confidence interval. Unadjusted model only included serum chloride which as qualitative data (<105.4 mmol/L and ≥105.4 mmol/L). Serum chloride < 105.4 mmol/L was reference. ^a^Model 1 was adjusted for sex and age. Sex was analyzed as dichotomous data, and the male was used as reference. ^b^Model 2 was adjusted for covariates in model 1 plus serum creatinine, 24-hour urine protein, and hypertension (yes or no), and no hypertension was used as reference. ^c^Model 3 was adjusted for covariates in model 2 plus Oxford classification grade M (mesangial hypercellularity), E (the presence of endocapillary proliferation), S (segmental glomerulosclerosis/adhesion), T (severity of tubular atrophy/interstitial fibrosis), and C (presence of crescent) scores. The latter five variables were analyzed as categorical data. M0/E0/S0/T0/C0 was used as references.

## Data Availability

Raw data used during the current study are available from the corresponding author on reasonable request for noncommercial use.

## Supplementary Materials

Supplementary Materials Supplemental Table 1: univariate Cox regression for screening out variates into predictive prognosis mode.
