# Supplementary material for: Elevated Serum Chloride Levels Contribute to a Poor Prognosis in Patients with IgA Nephropathy
Source: J Immunol Res. 2021 Oct 8;2021:3598135. doi: 10.1155/2021/3598135 (PMC8739901; doi:10.1155/2021/3598135)
Supplement: Supplementary Materials — Supplemental Table 1: univariate Cox regression for screening out variates into predictive prognosis mode. [file 3598135.f1.pdf]

Supplemental Material

Supplemental Table 1 univariate Cox regression for screening out variates into predictive prognosis models

	univariate Cox regression	
characters	OR(95%CI)	*P* value
Age(years)	1.01(0.99-1.04)	0.36
Sex	0.64(0.35-1.16)	0.14
Hypertension	2.97(1.62-5.45)	<0.001
24h-UTP（g/d)	1.12(1.04-1.21)	0.002
Urine RBC(/HP)	1.00 (1.00-1.001)	0.77
BUN(mmol/l)	1.18(1.13-1.23)	<0.001
sCr(umol/l)	1.01(1.01-1.01)	<0.001
UA(umol/l)	1.004(1.002-1.006)	<0.001
ALB(g/l)	0.99(0.96-1.03)	0.71
TCHO(mmol/l)	0.89(0.76-1.03)	0.12
TG(mmol/l)	1.00(0.87-1.16)	0.99
sC3(g/l)	0.97(0.76-1.23)	0.78
sC4(g/l)	0.95(0.55-1.63)	0.84
HGB(g/l)	0.96(0.95-0.98)	<0.001
K(mmol/l)	1.48(0.86-2.54)	0.15
Na(mmol/l)	1.02(0.93-1.11)	0.69
Serum chlorine group	3.22(1.76-5.86)	<0.001
Ca(mmol/l)	0.65(0.13-3.10)	0.58
Mg(mmol/l)	1.23(0.07-23.33)	0.89
P(mmol/l)	4.88(1.48-16.09)	0.009
M	2.17(1.21-3.90)	0.009
E	1.16(0.62-2.14)	1.16
S	3.64(1.54-8.59）	0.003
T		<0.001
T1	3.34(1.29-8.62)	0.01
T2	9.55(4.75-19.19)	<0.001
C		0.84
C1	1.19(0.65-2.17)	0.57
C2	1.23(0.29-5.17)	0.78
IgG		0.95
IgG(1)	1.11(0.47-2.61)	0.82
IgG(2）	1.20(0.30-4.98)	0.81
IgM		0.57
IgM(1)	1.02(0.52-2.00)	0.95
IgM(2)	1.69(0.74-3.85)	0.21
IgM(3)	0.000(0.000-?)	0.98
IgA		0.84
IgA(1)	1244.19(0.00-5.09E+082)	0.94
IgA(2)	3007.65(0.00-1.22E+083）	0.93
IgA（3）	3821.36（0.00-1.55E+083）	0.93

IgA（4）	0.99 (0.00-1.85E+278)	1.00
C3		0.21
C3(1)	0.75(0.29-1.90)	0.54
C3(2)	1.22(0.60-2.48)	0.58
C3(3)	2.35(0.88-6.33)	0.09
A		0.003
A(1)	1.98(0.67-5.89)	0.22
A(2)	4.15(1.74-9.91）	0.001
Tubular necrosis	0.91(0.22-3.77)	0.90

BUN, blood urine nitrogen; sCr, serum creatinine; UA, urine acid; ALB, albumin; 24h-UTP, 24hour-urine protein; Urine RBC, urine red blood cell;T-CHO, total cholesterol; TG, triglyceride; serum C3, serum complement 3; serum C4, serum complement 4; Hb, hemoglobin; K, potassium; Na, sodium; Ca, calcium; Mg, magnesium; P, phosphorus; For Oxford classification, mesangial cell proliferation score(M): M0 for score ⩽ 0.5, M1 for score > 0.5; endothelial cell hyperplasia (E): E0 for absent and E1 for present; segmental sclerosis or adhesion (S): S0 for absent and S1 for present; renal tubule atrophy or renal interstitial fibrosis (T): T0 for 25% renal tubule atrophy or renal interstitial fibrosis,T1 for 26%~50% and T2 ≥ 50%; crescentic lesions (C), C0 is no crescent, C1 is < 25% globular crescent, C2 is ≥ 25% globular crescent. For immunofluorescence, immunoglobulin G(IgG): 0 for -/+, 1 for +, 2 for ++; IgM, immunoglobulin M(IgM): 0 for -/+, 1 for +, 2 for ++, 3 for +++; immunoglobulin A(IgA): 0 for-/+, 1 for +, 2 for ++, 3 for +++, 4 for ++++; complement 3(C3)： 0 for-/+, 1 for +, 2 for ++, 3 for+++; the degree of vascular injury(A): 0 for no obvious abnormality, 1for simple vascular wall thickening, 2 for not only vascular wall thickening, but also other lesions, such as fibrinoid necrosis, vitreous degeneration and so on.; renal tubular necrosis: 0 for no necrosis, 1 for necrosis.
